# Biomechanical comparison of lumbar spine instability between laminectomy and bilateral laminotomy for spinal stenosis syndrome – an experimental study in porcine model

**DOI:** 10.1186/1471-2474-9-84

**Published:** 2008-06-11

**Authors:** Ching-Lung Tai, Pang-Hsing Hsieh, Weng-Pin Chen, Lih-Huei Chen, Wen-Jer Chen, Po-Liang Lai

**Affiliations:** 1 Graduate Institute of Medical Mechatronics, Department of Mechanical Engineering, Chang Gung University, Taoyuan, Taiwan; 2 Department of Orthopaedic Surgery, Chang Gung Memorial Hospital, Chang Gung University College of Medicine, Taoyuan, Taiwan; 3 Department of Biomedical Engineering, Chung Yuan Christian University, Chungli, 32023, Taiwan

## Abstract

**Background:**

The association of lumbar spine instability between laminectomy and laminotomy has been clinically studied, but the corresponding *in vitro *biomechanical studies have not been reported. We investigated the hypothesis that the integrity of the posterior complex (spinous process-interspinous ligament-spinous process) plays an important role on the postoperative spinal stability in decompressive surgery.

**Methods:**

Eight porcine lumbar spine specimens were studied. Each specimen was tested intact and after two decompression procedures. All posterior components were preserved in Group A (Intact). In Group B (Bilateral laminotomy), the inferior margin of L4 lamina and superior margin of L5 lamina were removed, but the L4–L5 supraspinous ligament was preserved. Fenestrations were made on both sides. In Group C (Laminectomy) the lamina and spinous processes of lower L4 and upper L5 were removed. Ligamentum flavum and supraspinous ligament of L4–L5 were removed. A hydraulic testing machine was used to generate an increasing moment up to 8400 N-mm in flexion and extension. Intervertebral displacement at decompressive level L4–L5 was measured by extensometer

**Results:**

The results indicated that, under extension motion, intervertebral displacement between the specimen in intact form and at two different decompression levels did not significantly differ (*P *> 0.05). However, under flexion motion, intervertebral displacement of the laminectomy specimens at decompression level L4–L5 was statistically greater than in intact or bilateral laminotomy specimens (*P *= 0.0000963 and *P *= 0.000418, respectively). No difference was found between intact and bilateral laminotomy groups. (*P *> 0.05).

**Conclusion:**

We concluded that a lumbar spine with posterior complex integrity is less likely to develop segment instability than a lumbar spine with a destroyed anchoring point for supraspinous ligament.

## Background

Decompression of the spinal canal is currently the standard treatment for degenerative lumbar spinal stenosis. However, studies have shown that total laminectomy increases segmental instability unless fusion is performed [1-4]. Various studies have proposed technical modifications of the standard laminectomy procedure, applicable to the cervical, thoracic and lumbar spines [5-7]. These techniques have evolved from attempts to adequately decompress spinal stenosis in patients while preserving spinal integrity. Technical variations of decompression for lumbar spinal stenosis include unilateral laminotomy, bilateral laminotomy, open-door type laminoplasty and laminectomy.

Clinical studies indicate that neurologic compression occurs most frequently at the level of the interlaminar window. Accordingly, this window has been used to afford adequate decompression by excision of ligamentum flavum, resection of laminar margins and trumpeted "undercutting" techniques of partial facetectomy while maintaining residual lamina, pars and facet joint stability. Neorological decompressions by bilateral laminotomy attempts to maintain spinal columnar stability by preserving the midline structures (spinous process, supraspinous and interspinous ligaments). However, if the midline structures are not partially removed, the trajectory for lateral decompression may be compromised [7,8]. Therefore, decompressing the ipsilateral foramen and lateral recess requires further facet joint resection, which compromises spinal stability.

A modified technique characterized by bilateral laminotomy and contralateral foraminotomy was described by Weiner [7]. To address the lateral recess osteophyte and ligamentum flavum, the trajectory of Kerrison rongeur crosses the midline under the well-preserved supraspinous ligament. By this procedure, the hypertrophy of the ligamentum flavum and osteophyte formations on the articular facets can then be removed without jeopardizing the integrity of the supraspinous ligament complex and facet joints. Although the clinical result of the Weiner technique is satisfactory, no previous studies have reported the advantages of this modified bilateral laminotomy from a biomechanical perspective. A detailed description of the biomechanical performance of this modified bilateral laminotomy may be helpful in clinical practice. Based on the clinical superiority of the modified bilateral laminotomy in the current study, a further biomechanical examination was performed to evaluate the mechanical behavior of the treated lumbar spine.

## Methods

### Specimen preparation

**Figure 1:**
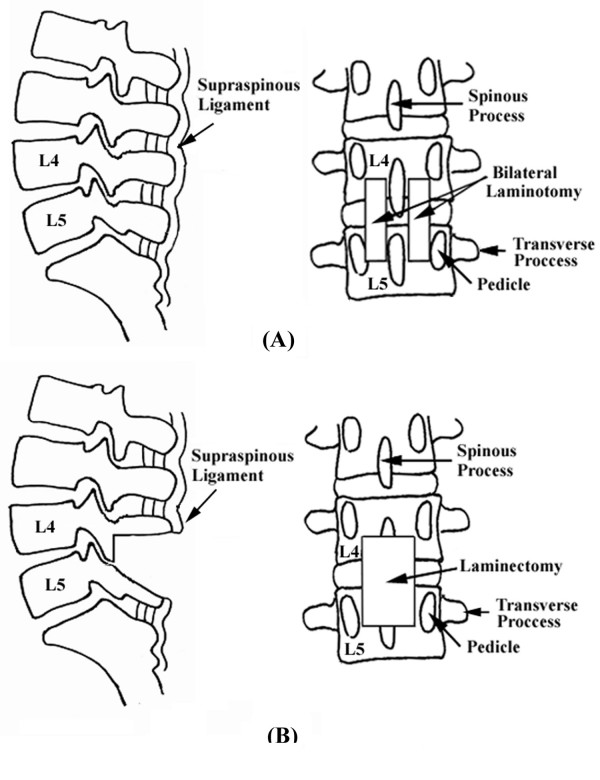
**Lateral View and posterior view of lumbar spine after (A) laminectomy and (B) bilateral laminotomy.** In laminectomy, the lamina and spinous processes of lower L4 and upper L5 are removed by rongeur and Kerrison clamp. The ligamentum flavum and supraspinous ligament of L4–L5 are removed. In laminotomy, fenestration is made on both sides. The inferior margin of L4 lamina and superior margin of L5 lamina are removed by burr. The L4–L5 ligamentum flavum is removed.

**Figure 2:**
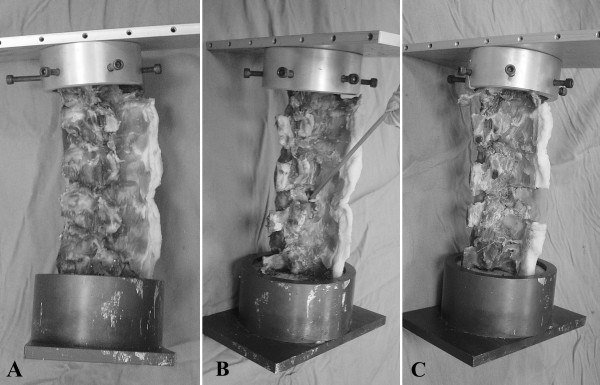
Photograph of the lumbar spine (A) intact, and at two different levels of decompression following (B) bilateral laminotomy and (C) laminectomy.

Eight adult porcine lumbar spines (L1–S1) were enrolled in this study. The paraspinal muscles of each specimen were completely excised, and all the ligamentous components, including the supraspinous ligaments, were carefully preserved. Each of the eight porcine lumbar spines was tested in intact form, after bilateral laminotomy and after laminectomy at different levels of decompressive surgery (Fig. 1 and Fig. 2). All posterior components were preserved in the intact group. In the bilateral laminotomy group, fenestration was made on both sides. The inferior margin of L4 lamina and superior margin of L5 lamina were removed by burr, and the L4–L5 ligamentum flavum was undercut. The L4–L5 supraspinous ligament was preserved in all specimens. Finally, in the laminectomy group, the lamina and spinous processes of lower L4 and upper L5 were removed by rongeur and Kerrison clamp. The ligamentum flavum and supraspinous ligament of L4–L5 were also removed. Preservation of bilateral L4–l5 facet joints was confirmed in all specimens.

### Biomechanical Test

**Figure 3:**
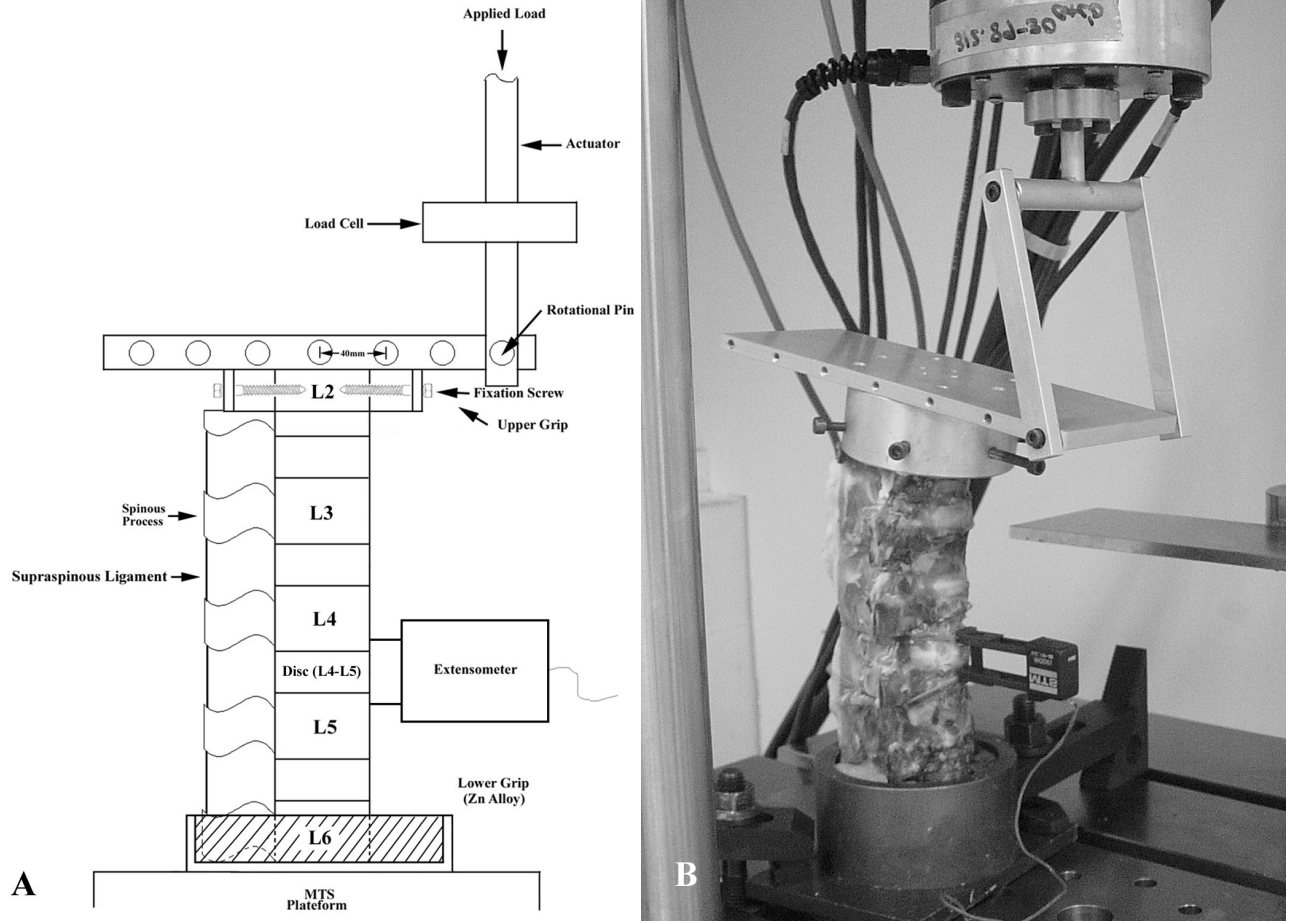
**Photograph of the (A) experimental setup for measuring intervertebral displacement and (B) flexion motion of intact lumbar spine.** By using a specially designed fixture, an 8,400 N-mm constant moment generated through the axial movement of the MTS actuator was applied to the spine specimen to achieve flexion and extension motions. Intervertebral displacement at L4–L5 adjacent to the fusion level was recorded continuously using an MTS extensometer.

The specimens were mounted for biomechanical testing by MTS (Bionix 858, MTS Corp., MN, USA). A specially designed fixture used to increase moment up to 8400 N-mm generated through the axial movement of the MTS actuator was applied to each specimen to achieve the flexion and extension motions. During testing, intervertebral displacement at decompression levels L4–L5 was recorded continuously by MTS extensometer (Model 632-12F-20, MTS Corp., US). Gauge length and resolution of the extensometer was 25.4 mm and 0.0127 mm, respectively. During testing, intervertebral displacement data were simultaneously recorded by MTS Testar II software. Six intervertebral displacement measurements of L4–L5 lumbar segment were performed in each porcine specimen. 1) Intact under flexion; 2) Intact under extension; 3) Bilateral laminotomy under flexion; 4) Bilateral laminotomy under extension; 5) Laminectomy under flexion; 6) Laminectomy under extension. For specimens with bilateral laminotomy, following the measurements of intact, bilateral laminotomy was performed by fenestration on both sides of lamina. The inferior margin of L4 lamina, superior margin of L5 lamina and the L4–L5 ligamentum flavum were removed. For specimens with laminectomy, following the measurements of bilateral laminotomy, laminectomy was performed by removal of the lamina and spinous processes of lower L4 and upper L5. The ligamentum flavum and supraspinous ligament of L4–L5 were also removed. All measurements were carried out using identical testing procedures. The stability of the lumbar spine in intact form, after bilateral laminotomy and after laminectomy were evaluated by comparing data for intervertebral displacement between L4–L5. Fig. 3 illustrates the experimental setup.

## Results

**Figure 4:**
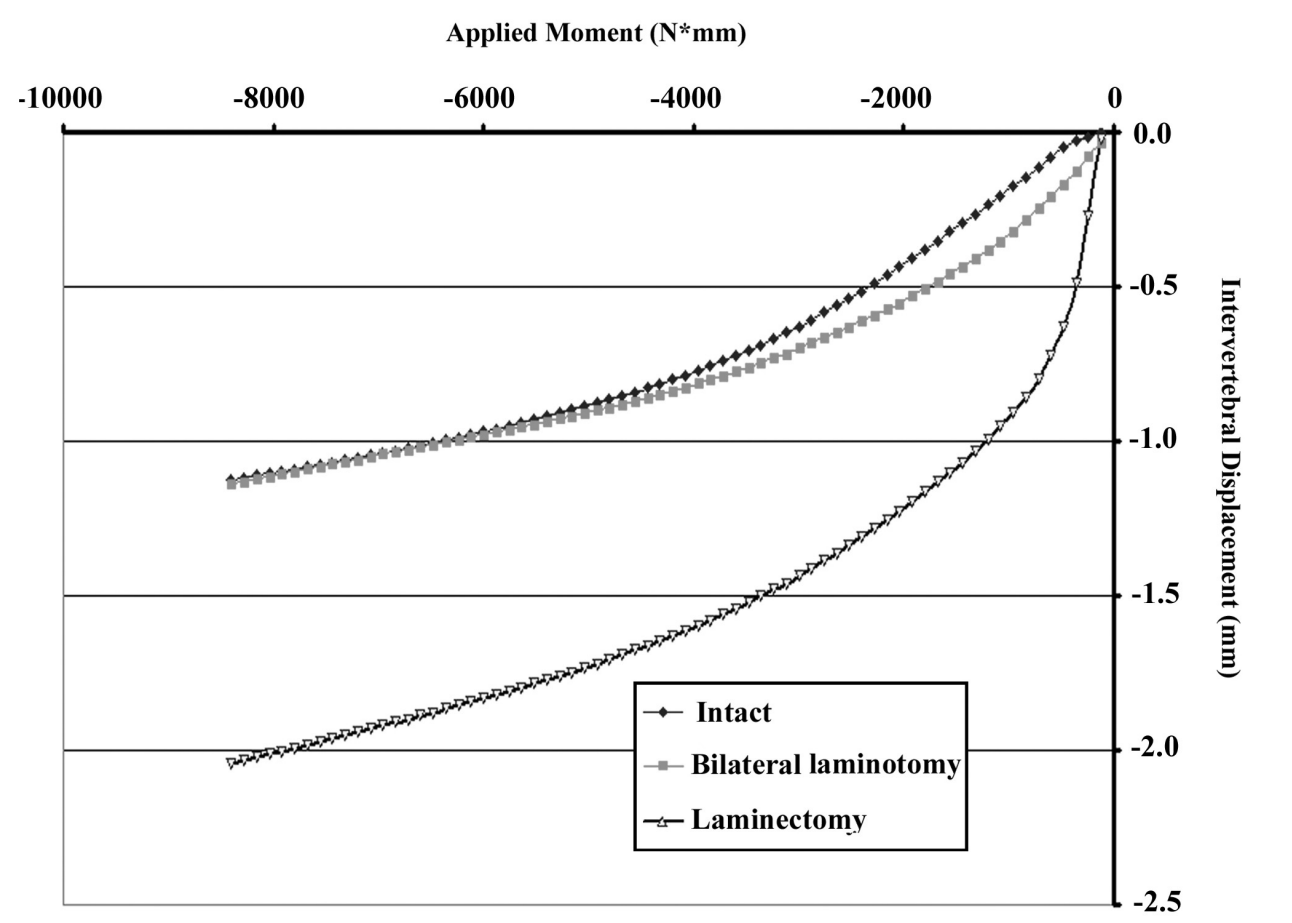
**A diagram of a typical intervertebral displacement versus applied moment on the decompressive segment under flexion motion.** The curve pattern demonstrates a significant decrease in intervertebral displacement of the decompressive segment as flexion moment increases in the early period. However, the rate (slope) gradually decreased as flexion moment increased.

Fig. 4 illustrates the typical intervertebral displacement versus applied moment on decompressive segment under flexion motion. The curve pattern demonstrates that the intervertebral displacement of the decompressive segment decreased significantly with the increasing flexion moment in the early period. However, the decreasing rate (slope) decreased gradually with increasing flexion moment.

**Figure 5:**
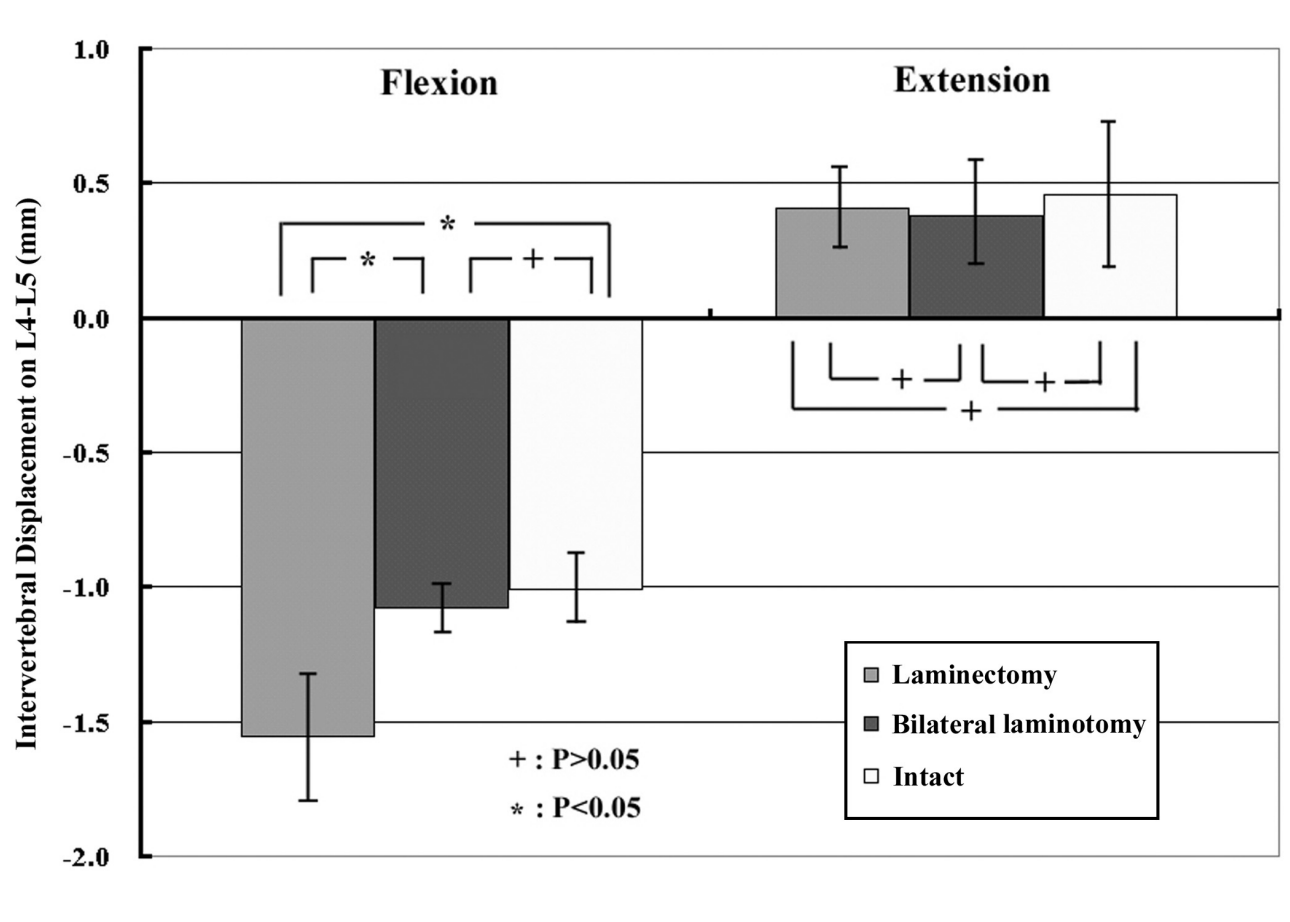
**Intervertebral displacement of L3–L4 segment under flexion and extension motions (8400 N-mm).** Intervertebral displacement between three different decompressive procedures under extension motion did not significantly differ (P > 0.05). However, under flexion motion, intervertebral displacement in the laminectomy group was statistically higher than in the intact or bilateral laminotomy groups (P < 0.05).

The maximal intervertebral displacement under conditions of 8400 N-mm were recorded for analysis. Table 1 and Fig. 5 display intervertebral displacements in decompression segment (L4–L5) for each of the three decompressive procedures under flexion and extension motions. The positive value represents the increased intervertebral displacement, while the negative value represents the decreased displacement. Theoretically, the higher the absolute value of change in disc height (either positive or negative), the less rigid the system, indicating a less stable overall spinal construct. Under extension motion, the facet joints locked and prevented posterior vertebral displacement. The moment increased rapidly to the end point of 8400 N-mm. The experimental results revealed no statistical difference in intervertebral displacement among the three different decompressive procedures (*P *> 0.05).

Under flexion motion, however, the intervertebral displacement of the decompression segment (L4–L5) with complete laminectomy was statistically greater than those of intact or bilateral laminotomy (*P *= 0.0000963 and *P *= 0.000418, respectively). Moreover, no difference was found between groups with intact and bilateral laminotomy (*P *> 0.05).

## Discussion

Spinal stenosis is a common disease, and several decompressive procedures for this condition are becoming more common in the practice of spinal surgery. Pathologic changes include bony spurs on the vertebral bodies, posterior protrusion of the intervertebral discs, hypertrophy of the ligamentum flavum and osteophyte formation on the articular facets [9-11]. Total laminectomy is currently the standard approach for degenerative lumbar spinal stenosis with a success rate reportedly as high as 85% to 90% [9,12]. However, the clinical effectiveness of laminectomy diminishes over time [13]. Many studies have shown that total laminectomy produces segmental instability unless fusion is performed [1-4]. Postacchini *et al *[12] compared multiple laminotomy and total laminectomy in sixty-seven clinical cases. Postoperative vertebral instability was rare in patients undergoing multiple laminotomy as compared with those who underwent total laminectomy. As the frequency of decompressive surgery for this condition increases, the ideal procedure will be one combining maximum canal and foraminal decompression with minimal resection of critical bony structures and supporting ligaments.

**Table 1:** The intervertebral displacements on L4–L5 segment for intact and two different extents of decompressive procedure. (Intact, bilateral laminotomy, and laminectomy). (Unit: mm)

	Intact		Bilateral laminotomy		Laminectomy	
	Flexion	Extension	Flexion	Extension	Flexion	Extension

Specimen 1	-1.118	0.944	-1.135	1.276	-2.015	1.187
Specimen 2	-1.116	0.992	-1.012	0.951	-1.433	0.860
Specimen 3	-1.123	1.033	-1.279	1.040	-1.338	0.938
Specimen 4	-0.892	0.710	-0.998	0.835	-1.402	0.944
Specimen 5	-1.004	0.829	-1.165	0.911	-1.410	1.155
Specimen 6	-.0832	0.955	-1.196	1.286	-1.675	1.023
Specimen 7	-1.238	0.873	-0.856	1.115	-1.864	0.968
Specimen 8	-0.984	0.892	-1.243	0.969	-1.473	0.815
Ave.	-1.038	0.904	-1.111	1.048	-1.576	0.986
S.D.	0.126	0.095	0.134	0.155	0.232	0.122

Most surgical approaches to decompression involve excision of the interspinous or supraspinous ligament complexes, altering an already pathologic biomechanical milieu. Goel *et al *[14,15] found that, under normal conditions, the supraspinous ligament received the greatest force when exposed to an external flexion moment across an anatomic segment. Kanayama *et al *[16] observed similar findings and suggested that, in regions lacking this ligamentous support, the paraspinal musculature provides compensatory stability. Resection of portions or all of the spinous processes, interspinous ligaments and supraspinous ligaments as well as iatrogenic damage to the paraspinal musculature excessively increases the volume of dead space [17]. Dead space and its consequent risks are significantly decreased using bilateral laminotomy instead of laminectomy. Preservation of the spinous processes and inter-/supraspinous ligamentous complex maintains the normal posterior median furrow, which would be lost using other more invasive techniques.

Numerous investigations have used the extensometer as an *in vitro *tool to test motion across discs after pedicle screw fixation [18-20]. Gurr *et al *[18] simulated spinal instability after anterior corpectomy for treatment of a fracture in a calf-spine model, and four types of posterior instrumentation were compared by measurement of intervertebral displacement across the corpectomy using an anterior extensometer. Shono *et al *[19] performed an *in vitro *biomechanical analysis of three anterior instability patterns on calf lumbosacral spines. Stiffness of the constructs and segment motion were compared by extensometer. Shono concluded that as segmental spinal instrumentation progresses from one level to three levels, overall rigidity of the system increases. Recently, Chen *et al *[20,21] investigated the effect of sagittal alignment on adjacent joint mobility after lumbar instrumentation in a porcine model. An anterior extensometer was used to compare the difference in intervertebral displacement of adjacent segments among instrumented lumbar spines in intact, lordotic and kyphotic alignment. The experimental results demonstrated that an instrumented spine in lordosis is less likely to develop adjacent instability than a kyphotic spine. In the present study, differences in intervertebral displacement between intact, bilateral laminotomy and laminectomy groups were compared by extensometer, and the relative differences of segmental instability after different decompressive procedures were thus examined.

In this study, the applied moment increased as applied loading increased. The maximal moment was preset at 8400 N-mm, which stopped the motion of flexion or extension when the moment reached the preset value of 8400 N-mm. Under extension motion, the facet joints locked and prevented posterior vertebral displacement, which caused the moment to increase rapidly to the end point of 8400 N-mm. This phenomenon was observed in all three groups. Under flexion motion, however, intervertebral displacement of the decompressive segment (L4–L5) in the laminectomy group was statistically greater than in the intact or bilateral laminotomy groups. Theoretically, this induces the greatest stress on the decompressive segment and leads to the degenerative acceleration of the decompressive segment following laminectomy despite preservation of the facet joint. The present results indicate that deconstruction of the anchor point for the supraspinous ligament might cause severe segmental instability after a decompressive procedure. The results also suggest that bilateral laminotomy is generally effective in lumbar spine decompressive surgery.

Although efforts have been made to simulate the clinical conditions, there are certain limitations to this study. First, under laboratory environment, this experimental study use porcine lumbar spine instead of human cadaveric spines. Although physiological structures such as spinal alignment, number of lumbar segments of the porcine spines are somewhat different from those of human cadaveric spines, however, animal spines are the most convenient choice to perform the experiment with long spinal segments on circumstance that human cadaveric spines can not be accessed. Numerous researches [22-28] have been done to evaluate the biomechanical behaviors of spinal column with use of porcine lumbar spines as a model for the human spine. Smit's report [22] had shown similarity in the mechanics of quadruped and human. He concluded that a quadruped can be a valuable model for the study of the spine in spite of its horizontal position. An important point of difference is the higher axial compression stress in quadrupeds, which leads to higher bone densities in the vertebrae. Second, unlike a constant bending moment across the spinal levels performed by Smith et al, who investigated the effects of surgical modification upon the canine lumbosacral spine using four-point bending [29], this study use an eccentrically applied compressive force to spinal segments, which results in a different load state at each spinal level as described in Kostuik and Smith [30]. From a biomechanical point of view, an applied eccentric load will lead to different loads at each spinal level since the load at an individual spinal level depend on the flexibility of the spine. For example, the applied moment will be proportional to the applied load and the moment arm, and this moment arm increases as the specimen flexibility increases. Therefore, the eccentrically applied moment will vary due to the change of specimen stiffness resulted from different surgical reconstruction procedures. Nevertheless, although the loading mode does not necessarily represent the actual physiological loading condition and may have great impact on the clinical relevance, the specimens in this study were all prepared and tested in a uniform and reproducible manner and we believe that these results provide useful information to orthopedic surgeons in performing decompressive surgery for lumbar spinal stenosis. Third, the loading conditions considered were only motion in saggital plane (flexion and extension). Further investigation on the effects of other loading conditions such as lateral bending and axial rotation might be necessary in the future.

## Conclusion

In this study, intervertebral displacement of the lumbar spine following laminectomy was significantly greater than that of the lumbar spine in intact form or following bilateral laminotomy. The abnormal stress on the decompressive segment causes spinal instability treated with laminectomy. Therefore, the integrity of posterior complex (spinous process-supraspinous ligament-spinous process) acts as a tension band in flexion and helps stabilize the decompressive spine. Destruction of the supraspinous ligament may jeopardize this stability of the decompression segment. If the integrity of the posterior complex is completely destroyed by the decompression approach, it is more likely to develop segmental instability. To conclude, bilateral laminotomy presents a more stable performance as compared to the traditional laminectomy in decompressive surgery.

## Competing interests

The authors declare that they have no competing interests.

## Authors' contributions

C–LT participated in the study design, in collecting the data, the statistically analyses and drafting of the manuscript. P–HH, W–PC, L–HC and W–JC participated in the study design. P–LL advised and assisted drafting of the manuscript. All authors read and approved the final manuscript.

## Pre-publication history

The pre-publication history for this paper can be accessed here:


